# Association of lysyl oxidase-like 1 gene common sequence variants in Greek patients with pseudoexfoliation syndrome and pseudoexfoliation glaucoma

**Published:** 2013-07-12

**Authors:** Ioanna Metaxaki, Pantelis Constantoulakis, Miltiadis Papadimitropoulos, Eftihia Filiou, Gerasimos Georgopoulos, Aggeliki Chamchougia, Dimitrios Papakonstantinou, Nikos Markomichelakis, Chrysanthi Koutsandrea, Ioannis Halkiadakis

**Affiliations:** 1 Ophthalmiatrion Athinon, Athens Eye Hospital, Athens, Greece; 2 LOCUS MEDICUS Diagnostic Center, Athens, Greece; 3 Department of Ophthalmology, Medical School, University of Athens, Greece; 4 Department of Applied Mathematics, University of Athens, Athens, Greece

## Abstract

**Purpose:**

Three common sequence variants in the lysyl oxidase-like 1 (*LOXL1*) gene were recently associated with pseudoexfoliation (PEX) and pseudoexfoliation glaucoma (PEXG) in populations from various parts of the world. In this study, the genetic association of these variants was investigated in Greek patients with PEX and PEXG.

**Methods:**

The three *LOXL1* single nucleotide polymorphisms (SNPs), one intronic (rs2165241) and two nonsynonymous coding SNPs (rs1048661: R141L and rs3825942: G153D), were genotyped in a total of 48 unrelated patients with PEX, 35 patients with PEXG, and 52 healthy subjects who had normal findings in repeated ophthalmic examinations. A genetic association study was performed.

**Results:**

Between the two coding SNPs, R141L did not show an association with PEX (p=0.297 for allele G, p=0.339 for genotype GG), whereas allele G of G153D showed a significant association (odds ratio [OR]=3.52, 95% confidence interval [CI]=1.735–7.166, p=3.24×10^−4^ for allele G, p=0.004 for genotype GG). Likewise, for the intronic SNP of rs2165241, genotype TT (p=0.005) and its corresponding allele T (OR=2.99, 95% CI=1.625–5.527, p=3.53×10^−4^) showed a significant association with PEX. The allele G of G153D showed a significant association with PEXG (OR=3.74, 95% CI=1.670–8.387, p=0.001). The combined haplotype GGT, consisting of all three risk alleles, was associated with PEX (p=0.037), conferring a 1.8-fold of increased risk to the disease (OR=1.799, 95% CI=1.04–3.13). Furthermore, the haplotype GGT presented in 39.8% of the patients with PEX and 26.9% of the controls.

**Conclusions:**

Certain genetic variants in *LOXL1* confer risk for PEX in Greek populations, confirming in part findings in patients from Northern Europe.

## Introduction

Pseudoexfoliation (PEX) is an age-related syndrome characterized by the accumulation of white, small deposits of fibrinogranular extracellular material in the anterior segment of the eye [1]. PEX is the most common identifiable risk factor for glaucoma accounting for approximately 20%–25% of cases. Patients with PEX are twice as likely to convert from ocular hypertension to glaucoma, and when glaucoma is present, it progresses more rapidly [2,3]. Moreover, there is increasing evidence of an etiological association of PEX with cataract formation, and possibly with retinal vein occlusion [4]. PEX is also suspected to be a systemic disorder and has been associated with transient ischemic attacks, stroke, systemic hypertension, coronary heart disease, and myocardial infarction [5-7].

The prevalence of PEX is higher among older individuals, and reported prevalence rates vary extensively from country to country and even within the same area. The prevalence of PEX ranges from 20% to 25% in Scandinavian countries to less than 1% in China and Japan [8-14]. The prevalence of PEX in Greece varies between 11.5% and 16% depending on the area [15,16].

A significant association between common single nucleotide polymorphisms (SNPs) in the lysyl oxidase-like 1 (*LOXL1*) gene on chromosome 15q24.1 with PEX and pseudoexfoliation glaucoma (PEXG) in populations of different ethnicity was recently established [17-36]. Three susceptibility SNPs have been detected among populations, one intronic (rs2165241) and two nonsynonymous coding SNPs (rs1048661: R141L and rs3825942: G153D). LOXL1 is a member of the lysyl oxidase family of enzymes that catalyze the covalent cross-linking of collagen and elastin in connective tissues through oxidative deamination of lysine or hydroxylysine side chains. The family is composed of five characterized members lysyl oxidase (LOX) and lysyl oxidase-like 1–4 (LOXL1–LOXL4). LOXL1 seems to be specifically required for tropoelastin cross-linking and has been shown to be involved in forming, maintaining, and remodeling elastic fiber [37,38].

From analysis of the PEX material, it has been proposed that PEX material arises from abnormal aggregation of elastin microfibrillar components (elastic microfibrillopathy) produced by various intraocular cell types. Although a role of LOXL1 in forming the extracellular matrix of the eye has not been documented, LOXL1 expression is detected in ocular tissues such as the lamina cribrosa, lens epithelium, cornea, ciliary muscle, and trabecular meshwork, all of which may be involved in forming the extracellular matrix [38,39]. The aim of the present study was to confirm the association of these SNPs in the *LOXL1* gene with PEX and PEXG in Greek patients.

## Methods

### Study subjects

Greek patients with clinically diagnosed PEX and PEXG and normal Greek controls were recruited at Athens Eye Hospital. Written informed consent was obtained from all subjects. The study protocol was approved the hospital’s ethics committee and was performed according to the tenets of the Declaration of Helsinki. All subjects underwent detailed ophthalmic examinations by ophthalmologists that included slit-lamp biomicroscopic examination, gonioscopy, dilated examination of the lens, and funduscopy. Subjects with PEX were defined as those with clinical evidence of pseudoexfoliation at the pupil margin, anterior lens surface, or other anterior segment structures with an intraocular pressure (IOP) of less than 21 mmHg and no clinical evidence of glaucomatous optic neuropathy. Subjects with PEXG were defined as those with clinical evidence of PEX and glaucomatous optic neuropathy (defined as loss of neuroretinal rim with a vertical cup:disc ratio greater than 0.7) with compatible visual field loss. Greek subjects from a cataract clinic with a normal anterior segment and optic nerve examination and without clinical signs of PEX or PEXG were recruited as controls.

### Blood collection, DNA isolation, and real-time polymerase chain reaction genotyping

Whole blood (2.5–5 ml) was drawn from every person included in the study, and genomic DNA was extracted from human peripheral blood nucleated cells using the QIAamp DNA Blood Mini kit (Qiagen, Hilden, Germany), following the manufacturer’s instructions. The DNA samples were stored in 100 μl of elution buffer at −40 °C. The three SNPs were genotyped with real-time PCR (LightCycler FastStart DNA Master HybProbe; Roche, Mannheim, Germany), followed by melting analysis. The primers and probes used are presented on Table 1.

**Table 1:** The primers and probes used in the present study

Polymorphism	PCR primers (5′ to 3′)	Probes (5′ to 3′)
R141L	GgggACAgCACTggCAT CggTAgTACACgAAACCCTggT	CATggCCCTggCCCgC—FL LC640-CCTCCgTCTCCCAgCAACggCAC
G153D	GCACCCATTCggCTTTg CggTAgTACACgAAACCCTggT	ACggggACTCCgCCTCCTC—FL LC640-gTCTCggCTTCggCCTTCgCCAg
rs2165241	CTgAgCTCTCAAATCCCA gCTTCCTTAAgAACTAACAgC	CCCACA XI ATACTggCAgAgg

XI represents modification with Inosin, FL represents fluorophore dye, LC640 represents LightCycler-red-640-n-hydroxysuccinimide-ester.

All primers and probes were stored at a concentration of 20 μM and 10 μM, respectively. In every reaction, 0.5 μM of each forward and reverse primer as well as 0.2 μM of the probes were used per 20 μl reaction. The MgCl_2_ concentration was 3.5 mM in R141L and G153D, whereas in the rs2165241 reaction 3.0 mM of MgCl_2_ was used_._ Finally, we used 0.5 μl of 2.5% DMSO in every reaction. The PCR conditions were the following: 95 °C for 10 min (denaturation), 95 °C for 10 s, 55 °C (50 °C for rs2165241) for 12 s, 72 °C for 10 s (45 cycles), 95 °C for 0 s, 40 °C for 30 s, 80 °C for 0 s (melting), 40 °C for 30 s (cooling).

The genotypes for all three SNPs were confirmed with direct genomic sequencing of a random group of samples. For sequencing, products from PCR amplification were purified and sequenced using ABI Big Dye chemistries and an automated ABI 3500 genetic analyzer, according to the manufacturer’s protocol (Applied Biosystems, Foster City, CA).

### Statistical analysis

Data were analyzed using PASW Statistics version 18.0.0 (SPSS Statistics Inc., New York, NY). The primary outcome variables were analyzed with contingency tables. Pearson's chi-square test was used to compare the patient (PEX/PEXG) and control groups for possible associations between SNP genotype, allele, and haplotype frequencies and disease state. Odds ratio (OR), relative risk, and confidence interval (CI) estimates were used to examine further interactions between the disease and specific alleles and genotypes. Hardy–Weinberg equilibrium (HWE) was performed to estimate haplotype frequencies, using Fisher exact tests. HWE and haplotype association analysis were assessed with Haploview version 4.2 software.

In addition, a two-tailed p value<0.05 represented statistical significance in hypothesis testing, and 95% confidence intervals were used.

## Results

The demographic data of the population studied are shown in Table 2. Allelic and genotype association testing results of the three *LOXL1* common sequence variants (rs2165241T–C, R141L G–T, and G153D G–A) in the patients with PEX and PEXG and the control subjects are shown in Appendix 1, Appendix 2, Appendix 3, and Table 3.

**Table 2:** Demographic data of patient population

Study population (n)	Age (years)	Sex
Controls (52)	76.77 (STD 6.25)	26 F/26 M
PEXG (35)	76.03 (STD 6.70)	13 F/22 M
PEX (48)	78.56 (STD 5.86)	29 F/19 M

PEX=pseudoexfoliation PEXG=pseudoexfoliative glaucoma

**Table 3:** Haplotype analysis results of rs1048661, rs3825942 and rs2165241 in LOXL1 for Greece cases with PEX and controls.

Haplotype	Cases PEX	Controls	OR (95%CI)	P value
GGT	39.8	26.9	1.799 (1.035–3.128)	0.037
GGC	15.9	16	0.997 (0.494–2.015)	0.994
GAT	9.7	10.9	0.879 (0.377–2.053)	0.766
GAC	7.1	17.6	0.356 (0.151–0.840)	0.015
TGT	10.6	5.9	1.901 (0.721–5.016)	0.188
TGC	11.5	12.6	0.901 (0.408–1.990)	0.797
TAT	2.7	1.7	1.595 (0.262–9.730)	0.61
TAC	2.7	8.4	0.297 (0.080–1.110)	0.057

The p values that presented indicate that GGT haplotype is nominally significant with a p value=0.037.

R141L did not show an association with PEX (p=0.297 for allele G, p=0.339 for genotype GG), whereas allele G of G153D showed a significant association (OR=3.52, 95% CI=1.735–7.166, p=3.24×10^−4^ for allele G, p=0.004 for genotype GG). Likewise, for the intronic SNP of rs2165241, genotype TT (p=0.005) and its corresponding allele T (OR=2.99, 95% CI=1.625–5.527, p=3.53×10^−4^) showed a significant association with PEX. The allele G of G153D showed a significant association with PEXG (OR=3.74, 95% CI=1.670–8.387, p=0.001) and with the combined PEX/PEXG group (OR 3.60, 95% CI=1.980–6.600; p=1.56×10^−5^). Similarly, the allele T of rs2165241 showed a significant association with combined PEX/PEXG (OR=1.85, 95% CI=1.117–3.058, p=0.016; Appendix 2). The allele G for G153D SNP confers 13 times higher risk for PEX and about four times for PEXG, whereas the T allele for rs2165241 confers five times higher risk for PEX (Appendix 3).

The combined haplotype GGT, consisting of all three risk alleles, was associated with PEX (p=0.037, nominally significant), conferring a 1.8-fold of increased risk of the disease (OR=1.8, 95% CI=1.03–3.28; Table 3).

## Discussion

The present study indicates that in a Greek population with PEX and PEXG, among the three identified SNPs a strong association was observed with the risk allele G153D. Furthermore, a weaker association with rs2165241T was observed in Greek samples of PEX. The results are consistent with the results of a recent ethnicity-based subgroup meta-analysis of the association of *LOXL1* polymorphisms with PEX [32]. According to the results of this meta-analysis, although each SNP investigated has been associated with PEX/PEXG through different studies and in different populations, the association of G153D with PEX/PEXG has been verified in most ethnic populations. Specifically, the OR of the G allele was 9.30 (95% CI=5.70–15.16, p<0.00001) in Caucasians. According to the present study, the OR of the G allele was 3.60 (95% CI=1.980–6.600, p=1.56×10^−5^) for the Greek population. Chen et al. [32] presented an OR of 7.05 for G153D under a dominant model (GG+GA versus AA) compared to OR=14.1, p=0.002, for PEX in the present study. In addition, Chen et al. presented OR=14.7 for a recessive model (GG versus GA+AA). This can be compared to OR=2.77 (p=0.017) in the present study (Appendix 4).

Furthermore, in accordance with previous studies, the present study identified an association between the high-risk T allele of the rs2165241 SNP and the PEX/PEXG group with an OR of 1.85. Subgroup meta-analysis [32] suggested that the OR of the T allele was 3.39 (95% CI=3.07–3.74, p<0.00001) in Caucasians ranging from 2.14 [18] to 4.52 [36] in different studies.

The present study identified no significant association between the high-risk G allele of the R141L SNP and the PEX/PEXG groups, similar to studies performed in Polish [24], Chinese, and Indian populations [32]. In contrast, studies analyzing a Japanese population have shown the reverse effect at this SNP, with the T allele as the risk allele [31]. Interestingly, the frequency of the risk G allele of R141L in the control group of the present study was surprisingly high (0.798) and appeared consistent among patients with PEX or PEXG, a finding that has not been described before.

Finally, the present study indicated that the combined haplotype GGT, consisting of all three risk alleles, presented in 39.8% of the patients with PEX, conferring a 1.8-fold of increased risk of the disease (95% CI=1.04–3.13). However, the association was nominally significant (p=0.037). This finding is different from Aragon-Martin et al.’s results [20] that showed in a cohort of American and European patients the GGT haplotype is overrepresented by 66% in patients with PEX.

The reasons underlying true variations in the prevalence of PEX and in the association of the SNPs investigated with PEX/PEXG, both from one ethnicity to another and within more or less homogenous populations, are not clearly understood and remain to be explained. Geographic distribution patterns may perhaps be explained either by regional gene pools or by environmental influences. Opposite risk alleles among populations and ethnic-related differences suggest that additional factors may contribute in PEX development. Similarly to the finding of opposite risk alleles observed in a Japanese population, the risk allele for a South African population for rs3825942 was A instead of the G allele found in Caucasian populations [33]. These findings question the hypothesis that these SNPs alone are responsible for PEX/PEXG and suggest that other genetic or environmental factors may predispose to exfoliation syndrome. Inconsistent genetic findings derived from different study populations led to an investigation of the effects of the *R141L* and G153D variations on amine oxidase activity of LOXL1. According to Kim et al. [40], the R141L and G153D variations in the NH_2_-terminal region of *LOXL1* do not affect the amine oxidase activity of LOXL1. It is more likely that these variations may play a role in directing LOXL1 onto the sites of elastogenesis by influencing its interaction with tropoelastin, or may affect the interaction of LOXL1 with fibulin-5, thus leading to disorganized formation and abnormal aggregation of elastic fibers.

Apart from *LOX L1*, several functional candidate genes involved in PEX material formation have been investigated in the past encoding fibrillin-1 (*FBN1*), latent TGF-β binding protein 2 (*LTBP2*), microfibril-associated protein 2 (*MFAP2*), transglutaminase 2 (*TGM2*), transforming growth factor β1 (*TGF-β1*), metalloproteinases (*MMPs*), their tissue inhibitors (TIMPs), and clusterin [41]. Specifically, *MMP1* and *MMP3* gene polymorphisms showed no clear-cut significant association with PEX syndrome and PEX glaucoma in Greek patients [42].

In conclusion, the present study shows that in a Greek population there is a strong association with G153D in the PEX and PEXG patient groups whereas in rs2165241 a strong association was seen only in patients with PEX. No association with R141L was seen in patients with PEX or PEXG. Opposite risk alleles among populations and ethnic-related differences suggest that additional factors may contribute in PEX development.

## Appendix 1. Allelic and genotype association of rs1048661, rs3825942, rs2165241 in PEX group.

Allelic and genotype association testing results of rs1048661, rs3825942 and rs2165241 in LOXL1 for Greek cases with PEX and controls are shown. The single asterisk indicates the p values, OR values and CI% for G versus T of rs1048661, G versus A of rs3825942 and T versus C of rs2165241, respectively. To access the data, click or select the words “Appendix 1.”

## Appendix 2. Allelic association of rs1048661, rs3825942, rs2165241 in PEXG and combined PEX/PEXG group.

Allelic association testing results of rs1048661, rs3825942 and rs2165241 in LOXL1 for Greek cases with PEXG and combined pseudoexfoliation syndrome and controls are shown. The single asterisk indicates the p values, OR values and CI% for G versus T of rs1048661, G versus A of rs3825942 and T versus C of rs2165241, respectively. To access the data, click or select the words “Appendix 2.”

## Appendix 3. Analysis of single genotypes of LOXL1 polymorphisms in Greek population.

The p values, OR values and CI% that are presented are for GG+GT versus TT of rs1048661, GG+GA versus AA of rs3825942 and TT+CT versus CC of rs2165241, respectively (dominant model). SNPs G153D and rs2165241 are associated with PEX even when the Bonferroni approach is used (both p values are less than 0.0056) whereas the association of G153 D with PEXG is nominally significant (p=0.031). PEX=pseudoexfoliation PEXG=pseudoexfoliative glaucoma. To access the data, click or select the words “Appendix 3.”

## Appendix 4. Allelic association of rs1048661, rs3825942 and rs2165241 in PEX, PEXG, combined PEX/PEXG group (recessive model).

Allelic association testing results of rs3825942 and rs2165241 in LOXL1 for Greek cases with PEX, PEXG, and combined pseudoexfoliation syndrome and controls are shown. The double asterisk indicates the p values, OR values and CI% for GG versus GA+AA of rs3825942 and TT versus CT+CC of rs2165241, respectively (recessive model). To access the data, click or select the words “Appendix 4.”
